# MRI tractography reveals the human olfactory nerve map connecting the olfactory epithelium and olfactory bulb

**DOI:** 10.1038/s42003-022-03794-y

**Published:** 2022-09-06

**Authors:** Sho Kurihara, Masayoshi Tei, Junichi Hata, Eri Mori, Masato Fujioka, Yoshinori Matsuwaki, Nobuyoshi Otori, Hiromi Kojima, Hirotaka James Okano

**Affiliations:** 1 Department of Otorhinolaryngology, The Jikei University School of Medicine, 3-25-8 Nishishimbashi Minato-ku, Tokyo, 105-8471 Japan; 2 Division of Regenerative Medicine, The Jikei University School of Medicine, 3-25-8 Nishishimbashi Minato-ku, Tokyo, 105-8471 Japan; 3 Graduate School of Human Health Sciences, Tokyo Metropolitan University, 7-2-10 Higashi-Ogu Arakawa-ku, Tokyo, 116-8551 Japan; 4 Department of Molecular Genetics, Kitasato University School of Medicine, 1-15-1 Kitasato Minami-ku Sagamihara-shi, Kanagawa, 252-0373 Japan; 5 Department of Otorhinolaryngology, Head and Neck Surgery, Keio University School of Medicine, 35 Shinanomachi Shinjuku-ku, Tokyo, 160-8582 Japan

**Keywords:** Olfactory bulb, Neural circuits, Olfactory bulb, Diffusion tensor imaging

## Abstract

The olfactory nerve map describes the topographical neural connections between the olfactory epithelium in the nasal cavity and the olfactory bulb. Previous studies have constructed the olfactory nerve maps of rodents using histological analyses or transgenic animal models to investigate olfactory nerve pathways. However, the human olfactory nerve map remains unknown. Here, we demonstrate that high-field magnetic resonance imaging and diffusion tensor tractography can be used to visualize olfactory sensory neurons while maintaining their three-dimensional structures. This technique allowed us to evaluate the olfactory sensory neuron projections from the nasal cavities to the olfactory bulbs and visualize the olfactory nerve maps of humans, marmosets and mice. The olfactory nerve maps revealed that the dorsal-ventral and medial-lateral axes were preserved between the olfactory epithelium and olfactory bulb in all three species. Further development of this technique might allow it to be used clinically to facilitate the diagnosis of olfactory dysfunction.

## Abstract (web summary)

Combined high-field MRI and DTI analyses in post-mortem mouse, marmoset, and human samples provide insight into the neural connections between nasal cavities and olfactory bulbs.

## Introduction

The first step in olfaction involves the binding of volatile small molecules to olfactory receptors on olfactory neurons in the nasal cavity. The chemical stimuli are transduced into electrical signals, and the projections of the olfactory neurons form nerve bundles that pass through the cribriform plate to reach the olfactory bulb, which is the primary center of olfaction. The surface of the olfactory bulb contains numerous glomeruli, and nerve fibers from olfactory neurons expressing the same olfactory receptor gene gather in a particular glomerulus^1^. Olfactory dysfunction can be caused by a variety of factors and is classified into three categories according to the anatomical location of the lesion: conductive (nasal disorders such as obstruction by polyps or inflammation), sensorineural (dysfunction of the olfactory sensory neurons themselves) and central (secondary to head trauma or conditions affecting the central nervous system such as brain tumors or neurodegenerative diseases). Clarifying the distribution of the olfactory sensory neurons in the nasal cavity and the projections of these nerves to the olfactory bulb are essential to understanding the pathogenesis of olfactory dysfunction.

Most studies exploring the distribution of the olfactory nerves in the nasal cavity and the neuronal pathways projecting to the olfactory bulb have been conducted in rodents. These investigations have included a histological analysis of the types of epithelial tissue lining the nasal cavity^2^, the use of transgenic animal models to visualize olfactory nerves expressing a single type of olfactory receptor^1^, and mapping of nerve pathways using neural tracers^3^. Previous findings in mice have revealed a topographical correlation between the nasal cavity and olfactory bulb known as the odor map^3^. However, the neural pathways running from the nasal cavity to the olfactory bulb in humans have not been characterized in detail, and various theories exist regarding the distribution of olfactory mucosal epithelium in the human nasal cavity. Additionally, the conventional techniques (e.g., neural tracers, immunostaining and transgenic animal models) used to evaluate the distribution of olfactory nerves are labor-intensive and require the use of tissue specimens to assess the continuity of nerves^4^, which precludes their utilization in large numbers of people or animals. Therefore, we conceived a new method of visualizing olfactory nerves using diffusion tensor tractography (DTT), which is based on magnetic resonance imaging (MRI). The development and refinement of DTT during the past few decades has been made possible by advances in MRI technology (including increases in magnetic field intensity) and improvements in analytical techniques. DTT virtually reconstructs neuronal pathways in three dimensions based on the diffusion and anisotropy of water molecules, and this technique is now commonly used in the field of neuroscience to estimate fiber pathways within the brain^5^.

In this study, we used mice, marmosets and humans to perform DTT on olfactory nerves within the nasal cavity and olfactory bulb. As mentioned above, most studies of olfactory nerves have used rodents, which have rather different nasal features to humans (including the number of ethmoturbinates, sinuses and olfactory receptor genes). On the other hand, the nasal features of marmosets share similarities with those of humans, and marmosets have gained attention in recent years as a non-human primate model^6^. Therefore, we first verified the validity of DTT using specimens from mice. Then, we carried out DTT and histological analyses using common marmosets. Finally, we performed DTT using human specimens. Our findings reveal the neuronal pathways in the nasal cavity and the destinations of their projections to the olfactory bulb in humans. Furthermore, comparisons between humans, marmosets and mice showed that the olfactory nerve projection patterns were preserved across species. Differences between mice and primates (marmosets and humans) were also noted, especially regarding the ethmoturbinal structures. Mice had more complex ethmoturbinal structures than primates, and the olfactory nerve fibers were more heavily distributed to the turbinal side than to the septal side in rodents but distributed more evenly between the two sides in primates. To our knowledge, this study is the first to generate the olfactory nerve maps of mice, marmosets and humans using DTT. Notably, DTT can be applied to the living brain, in contrast to the histological techniques and transgenic animal models used previously to create the olfactory nerve map of the mouse. We believe that DTT could provide valuable findings in basic medical research and be applied clinically as an objective method to evaluate the olfactory nerves.

## Results

### The distribution of mouse olfactory nerves revealed by DTT was comparable to that described in previous studies using different techniques

**Fig. 1:**
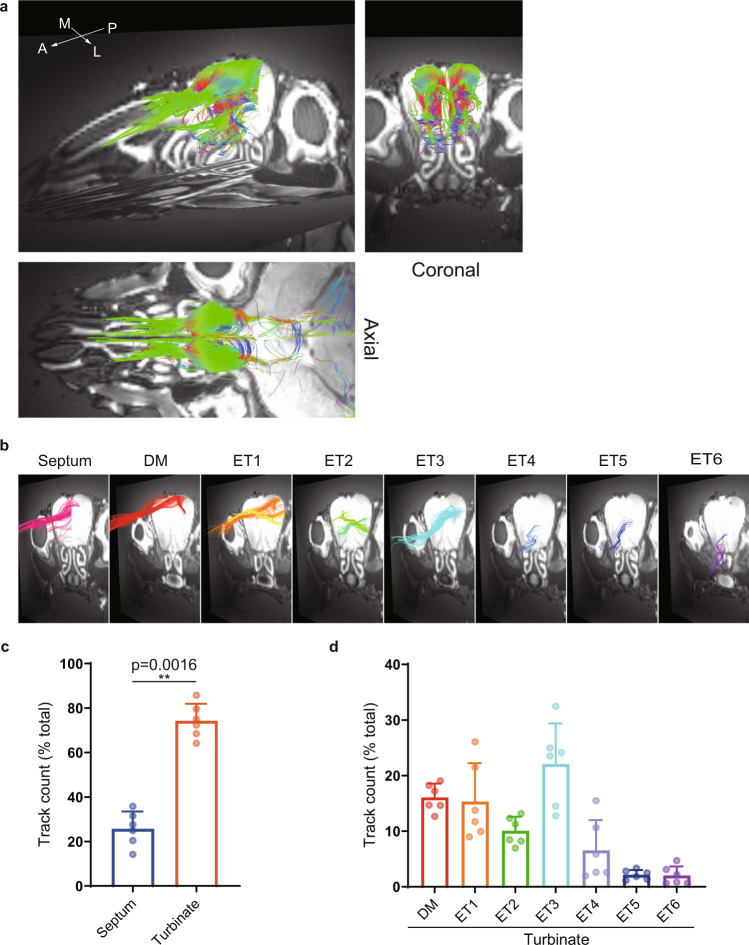
The distribution of mouse olfactory nerves depicted by diffusion tensor tractography (DTT). **a** DTT was performed using the whole skull of a mouse. The reconstructed three-dimensional images show oblique-sagittal, coronal and axial views. The colored fibers were visualized with DTT, and b0 images were used as the background images. The olfactory bulbs were selected as the region of interest (ROI) so that the depicted fibers reflected the neurons that projected from the nasal cavity to the olfactory bulbs. The fibers are color-coded according to their directionality: anterior-posterior axis, green; medial-lateral axis, blue; and superior-inferior axis, red. A anterior, L lateral, M medial, P posterior. **b** Oblique-sagittal views of the fiber tracts projecting from the main anatomical structures in the nasal cavity. The nerve tracts identified by DTT were color-coded according to the structure selected as the ROI (septum, dorsal meatus or each of the six ethmoturbinates). Each b0 image represents a coronal cross-section of the oblique-sagittal image in Fig. 1a. DM dorsal meatus, ET ethmoturbinate. **c** The track counting function of TrackVis was used to measure the density of fibers distributed in the septum and turbinates. Each value is expressed as the percentage of the total track counts. Bars show the mean and standard deviation. Individual data points are shown in dots (*n* = 6; 6 nasal cavities from 3 biologically independent specimens). **p < 0.01. **d** The percentage of all track counts of the fibers distributed to the dorsal meatus and each of the six turbinates (ET1–6). Bars show the mean and standard deviation. Individual data points are shown in dots (*n* = 6; 6 nasal cavities from 3 biologically independent specimens).

First, we examined the projection of nerve fibers from the nasal cavity to the olfactory bulb by selecting regions of interest (ROIs) on the olfactory bulbs of the mouse (Fig. 1a). Nerve fibers projected from the nasal septum to the medial part of each olfactory bulb and from the ethmoturbinates to the lateral part of each olfactory bulb. The distributions of nerve fibers in the nasal cavities and the neural projections from the nasal cavities to the olfactory bulbs were similar to those reported in transgenic mice in which olfactory marker protein (OMP) was replaced by tau-lacZ^1^ (Supplementary Fig. 1), suggesting that the fibers depicted by DTT were olfactory nerves. Subsequently, ROIs were positioned at different anatomical structures in each nasal cavity (septum, dorsal meatus and each of the six ethmoturbinates). The resulting tracts are shown in Fig. 1b. Data obtained from the left and right nasal cavities of three mice were used to compare track counts between the various anatomical structures (the track density was measured for each structure using the same DTT settings and then expressed as a proportion of the total track density for all structures). Track density was lower in the septum than in the turbinates (which have a more complex structure), indicating stronger nerve connectivity and distribution towards the turbinates (Fig. 1c, d).

**Fig. 2:**
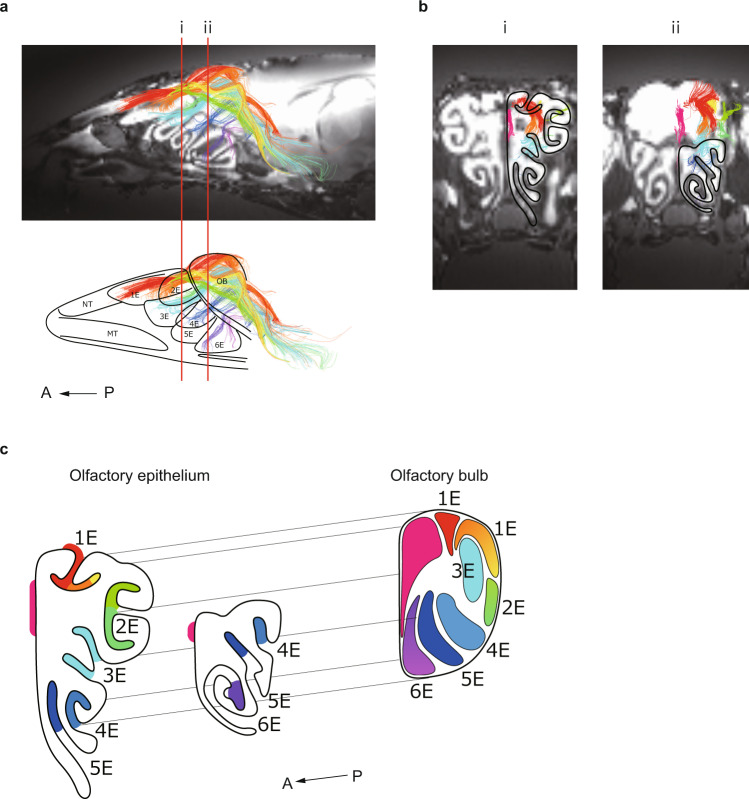
The topographical relationships of the olfactory nerves running between the olfactory epithelium and olfactory bulb of the mouse. **a** Sagittal sections depicting the nerve pathways projecting from the olfactory epithelium to the olfactory bulb. The fibers are color-coded according to their distribution in the olfactory epithelium (more than one region of interest was selected at some sites, such as the first, second and sixth ethmoturbinates). 1E–6E, first to sixth ethmoturbinate; A anterior, MT maxilloturbinate, NT nasoturbinate, OB olfactory bulb, P posterior. **b** Coronal sections of the same nerves depicted in Fig. 2a. i: rostral, ii: caudal. The nerve fibers were aligned in the medial-lateral axis. **c** Schematic diagram showing the DTT-based olfactory nerve map for the mouse (the distribution of the olfactory nerves running between the olfactory epithelium and olfactory bulb). Topographically, there was a correlation between the distributions in the olfactory epithelium and olfactory bulb in both the dorsal-ventral and medial-lateral axes. 1E–6E, first to sixth ethmoturbinate; A anterior, P posterior.

Next, we analyzed the nerve pathways between the olfactory epithelium and olfactory bulb. Figure 2a shows the neural pathways depicted from a lateral perspective, with the olfactory nerves color-coded according to the anatomical sites described in Fig. 1b. Notably, the olfactory nerves were widely distributed along the six ethmoturbinates. The same nerves are depicted in coronal sections (i: rostral, ii: caudal) in Fig. 2b, and a rotating three-dimensional (3D) reconstruction is shown in Supplementary Movie 1. Analysis of the distribution of each color-coded nerve (shown schematically in Fig. 2c) revealed a topographical correlation between the olfactory epithelium and olfactory bulb along the dorsal-ventral and medial-lateral axes. Although nerves from the second ethmoturbinate (2E) appear to intersect with those from 3E in the lateral perspective, the anterior perspective shows that the nerves do not intersect but exhibit a medial-lateral relationship. The topographical relationships identified by DTT are consistent with those described by previous studies using alternative methods^3,7,8^, which supports the validity of our approach. Miyamichi et al.^7^ reported that class 1 olfactory receptors^9^ are distributed in 3E and the dorsal meatus, and Bozza et al.^10^ reported that class 1 olfactory receptors are expressed on the dorsal side of the olfactory bulb. Our DTT-based analyses indicated that nerves from 3E and the dorsal meatus projected to the dorsal area of the olfactory bulb, which is consistent with the results of these previous reports.

### Use of DTT to depict the distribution of marmoset olfactory nerves

**Fig. 3:**
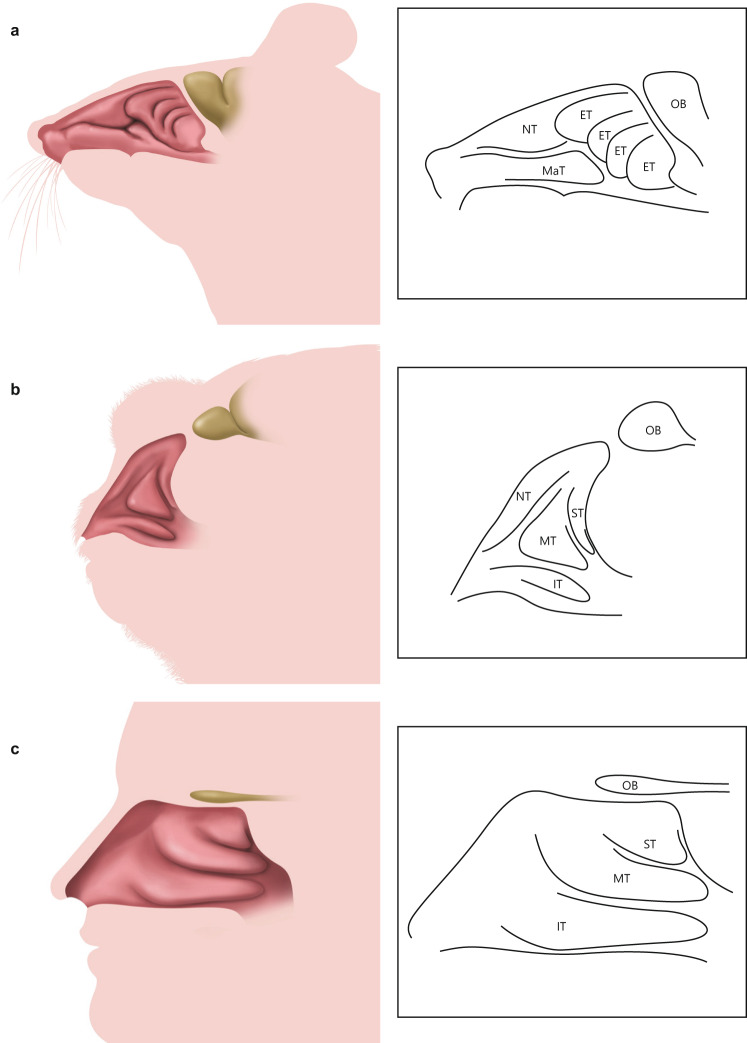
Schematic drawings of the nasal structures of the mouse, common marmoset and human. **a** Mouse. **b** Common marmoset. **c** Human. ET ethmoturbinate, IT inferior turbinate, MaT maxilloturbinate, MT middle turbinate, NT nasoturbinate, OB olfactory bulb, ST superior turbinate.

Although our ultimate aim was to validate the use of DTT as a method of assessing olfactory nerve distribution in humans, this objective was hampered by three main limitations: (1) specimens obtained from donated bodies were only available from an elderly population; (2) the donated bodies were fixed in formalin a few days after death and stored by formalin immersion, making them unsuitable for immunohistological analysis; and (3) the tissue required substantial trimming for it to fit into the experimental MRI machine. Therefore, DTT was used to evaluate the olfactory nerve distribution in young marmosets, with a small number of additional experiments carried out using human specimens. Marmosets are small primates with similar nasal structures^11^ and odorant receptor numbers^12^ to humans. Figure 3 shows schematic drawings of the nasal structures in mice, marmosets and humans. All three species have a single maxilloturbinate and multiple ethmoturbinates, although the number of ethmoturbinates differs between mice (six) and marmosets or humans (two). Since marmosets and humans have a similar turbinate structure, this article utilizes the same terminology for marmosets as that commonly used for humans (superior turbinate and middle turbinate).

**Fig. 4:**
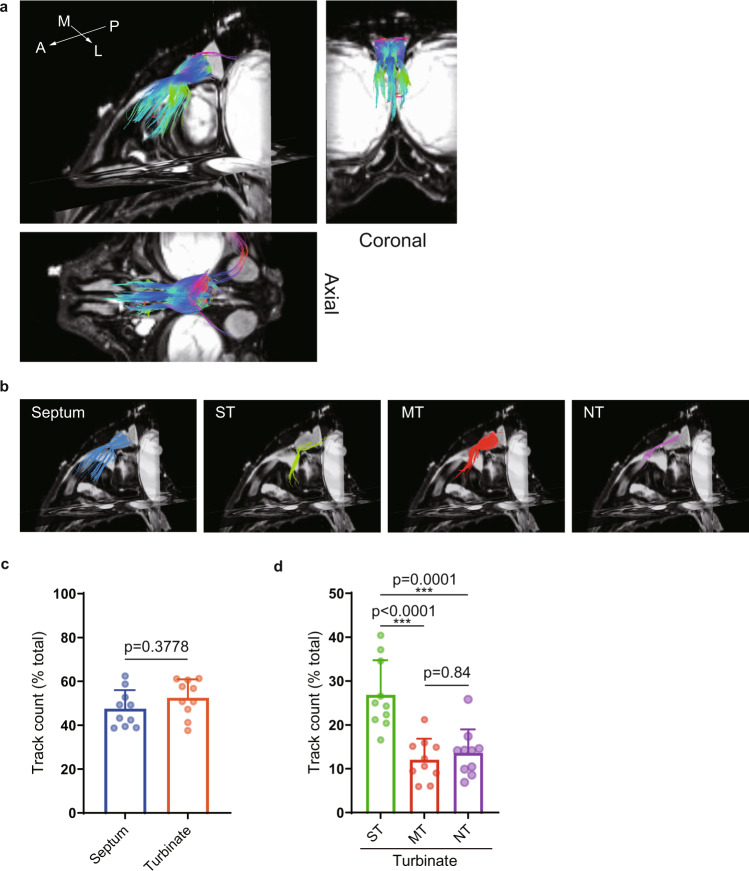
The distribution of marmoset olfactory nerves depicted by diffusion tensor tractography (DTT). **a** DTT was performed using the whole skull of a marmoset. The reconstructed three-dimensional images show oblique-sagittal, coronal and axial views. The colored fibers were visualized with DTT, and b0 images were used as the background images. The olfactory bulbs of the marmoset were set as the region of interest (ROI) so that the depicted fibers reflected the neurons projecting from the nasal cavity to the olfactory bulbs. The fibers are color-coded according to their directionality: anterior-posterior axis, blue; medial-lateral axis, red; and superior-inferior axis, green. A anterior, L lateral, M medial, P posterior. **b** Oblique-sagittal views of the fiber tracts projecting from the main anatomical structures in the nasal cavity. The nerve tracts identified by DTT were color-coded according to the structure selected as the ROI (septum, middle turbinate, nasoturbinate or superior turbinate). MT middle turbinate, NT nasoturbinate, ST superior turbinate. **c** The track counting function of TrackVis was used to measure the density of fibers distributed in the septum and turbinates. Each value is expressed as the percentage of the total track counts. Bars show the mean and standard deviation. Individual data points are shown in dots (*n* = 10; 10 nasal cavities from 5 biologically independent specimens). **d** The percentage of all track counts of the fibers distributed to each of the turbinates. MT middle turbinate, NT nasoturbinate, ST superior turbinate. Bars show the mean and standard deviation. Individual data points are shown in dots (*n* = 10; 10 nasal cavities from 5 biologically independent specimens). ***p < 0.001.

Figure 4a depicts fibers projecting from the nasal cavity to the olfactory bulb of the marmoset when the left olfactory bulb was set as the ROI. The fibers were distributed to four main anatomical structures: nasal septum, superior turbinate, middle turbinate and nasoturbinate. Figure 4b shows the fiber distribution when each of these four anatomical structures in the nasal cavity was selected as the ROI. Calculation of the track density for each anatomical structure using five marmoset specimens (track counting was performed bilaterally) suggested that there was a similar connectivity and distribution of nerve fibers to the septal and turbinal sides (Fig. 4c) and a stronger connectivity and distribution of nerve fibers towards the superior turbinate than towards the middle turbinate or nasoturbinate (Fig. 4d).

**Fig. 5:**
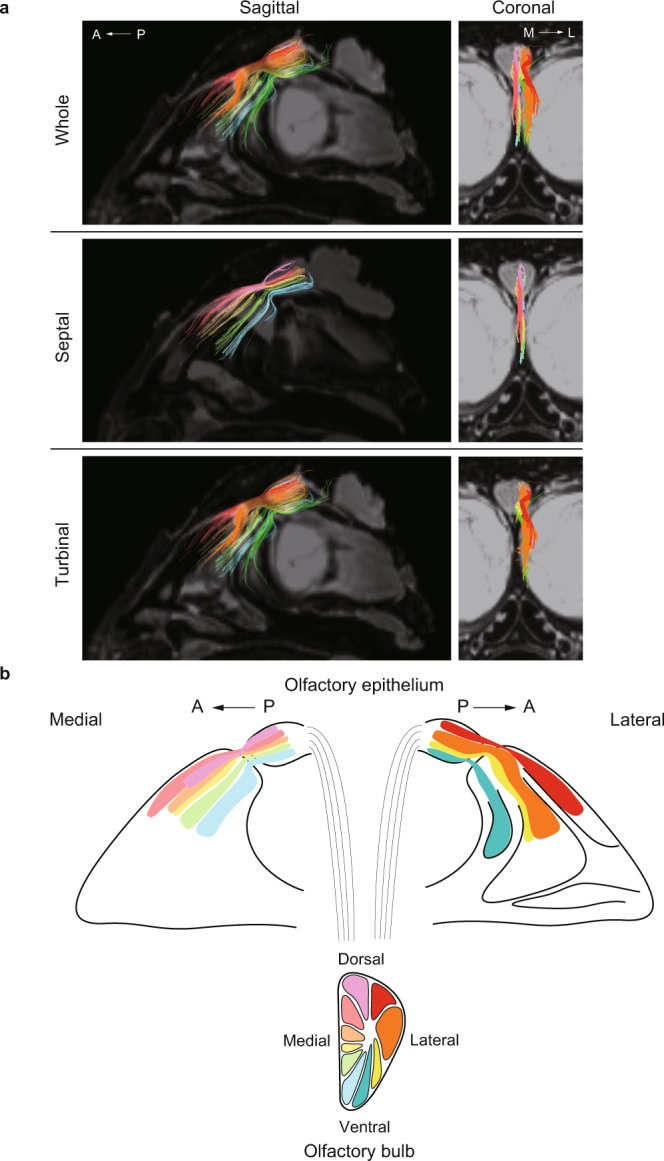
The topographical relationships of the olfactory nerves running between the olfactory epithelium and olfactory bulb of the common marmoset. **a** Sagittal and coronal sections depicting the nerve pathways from the olfactory epithelium to the olfactory bulb. The fibers are colored separately according to each olfactory filament. The Whole panels show all the depicted fibers, while the Septal and Turbinal panels show the septal and turbinal fibers, respectively. A anterior, L lateral, M medial, P posterior. **b** Schematic diagram showing the DTT-based olfactory nerve map for the marmoset (the distribution of the olfactory nerves running between the olfactory epithelium and olfactory bulb). Topographically, there was a correlation between the distributions in the olfactory epithelium and olfactory bulb in both the dorsal-ventral and medial-lateral axes. A anterior, P posterior.

Next, the ROI was set to each of the identifiable olfactory filaments (olfactory nerve bundles passing through the cribriform plate) in order to reveal the projection pathways of the marmoset olfactory nerves in more detail. The fibers distributed from each ROI are shown in different colors in Fig. 5a. Analysis of the projection pathways confirmed that nerves from the medial (septal) side of the nasal cavity projected to the medial portion of the olfactory bulb, while nerves from the lateral (turbinal) side of the nasal cavity projected to the lateral part of the olfactory bulb. Additionally, both the septal and turbinal sides of the nasal cavity exhibited a topographical correlation with the olfactory bulb along the dorsal-ventral axis. A rotating 3D reconstruction is presented in Supplementary Movie 2. Based on the above data, we were able to generate an olfactory nerve map for the common marmoset (Fig. 5b).

### Histological analyses of marmoset olfactory nerves

Paraffin-embedded tissue sections were prepared after the completion of MRI scanning in order to compare the marmoset olfactory nerve distribution obtained using DTT with that obtained by histological analysis. Olfactory nerve cells and olfactory epithelium were detected by immunostaining of OMP, and the dorsal region of the olfactory epithelium was identified by immunostaining of acyl-coenzyme A synthetase medium chain family member-4 (ACSM4)^13^.

**Fig. 6:**
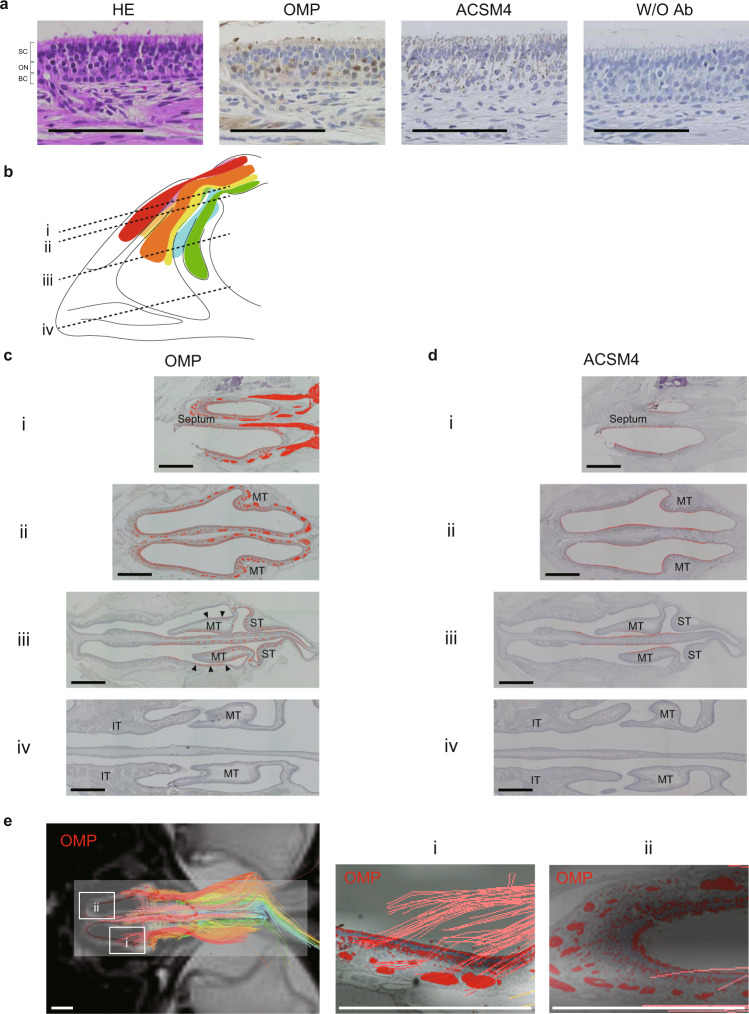
Histological sections of marmoset tissue immunostained for olfactory marker protein (OMP) or acyl-coenzyme A synthetase medium chain family member-4 (ACSM4). **a** Histological sections of the olfactory epithelium of the marmoset. The mucosa consists of an upper layer containing mainly supporting cells (SC), a middle layer of olfactory nerve cells (ON) that display chemoreceptors on the mucosal surface, and a lower layer of basal cells (BC). Nerve bundles consisting of multiple nerve cell axons can be seen in the submucosa. Scale bars: 100 μm. ACSM4, acyl-coenzyme A synthetase medium chain family member-4; HE, hematoxylin-eosin; OMP, olfactory marker protein; W/O Ab, without antibody. **b** Tissue sections were obtained at four levels (i, ii, iii and iv) for further analysis. **c** Histological sections of the marmoset nasal cavity immunostained for OMP. The OMP-positive regions are shown in red. OMP-positive regions were seen in both the mucosal layer and as nerve bundles in the submucosal layer. Scale bars: 1 mm. IT inferior turbinate, MT middle turbinate, OMP olfactory marker protein, ST superior turbinate. **d** Histological sections of the marmoset nasal cavity immunostained for ACSM4. The ACSM4-positive regions are highlighted in red. The ACSM4-positive regions were limited to the mucosal layer and were less extensive than the OMP-positive regions. Scale bars: 1 mm. IT inferior turbinate, MT middle turbinate, OMP olfactory marker protein, ST superior turbinate. **e** Overlaid magnetic resonance images, tractography images and OMP-stained images. Tractography detected the thicker nerve bundles in the submucosal layer. Scale bars: 1 mm.

First, each antibody was validated using sections of nasal septum epithelium. Staining for OMP was positive in olfactory nerve cells including their fibers, and ACSM4 was expressed in olfactory nerve cells, supporting cells and basal cells (Fig. 6a). Subsequently, the expressions of OMP and ACSM4 were examined in tissue sections obtained at four different levels (i–iv in Fig. 6b and Supplementary Fig. 2a). OMP was distributed in the superior turbinate, middle turbinate and superior portion of the nasal septum (Fig. 6c and Supplementary Fig. 2b). Interestingly, OMP was also expressed in the lateral epithelium of the middle turbinate (arrowheads in Fig. 6ciii). ACSM4 was expressed in some of the OMP-positive regions, the anterior portion of the nasal septum and the medial epithelium of the middle turbinate (Fig. 6d and Supplementary Fig. 2c). By referring to the olfactory nerve map in Fig. 5b, it may be seen that the ACSM4-positive region of the nasal cavity corresponded to the dorsal side of the olfactory bulb. This distribution pattern is consistent with that seen in mice^11^.

Next, OMP-stained tissue sections were overlapped with corresponding DTT images (with the olfactory bulb set as the ROI). The fibers depicted by DTT were well matched with the thick nerve fiber bundles of OMP-positive olfactory nerve cells (Fig. 6ei). On the other hand, thin olfactory nerve fibers were not depicted by DTT (Fig. 6eii), suggesting that the actual distribution of olfactory nerves may be wider than that depicted by DTT.

### Use of DTT to depict the distribution of human olfactory nerves

We used two donated human specimens (obtained from a 79-year-old male and an 81-year-old female) to visualize the distribution of human olfactory nerves by DTT. Since the bore diameter of the MRI device was only 90 mm, we dissected out the nasal cavity and the cribriform plate with the olfactory bulb still attached (to maintain the integrity of the neuronal connections between the nasal cavity and olfactory bulb).

**Fig. 7:**
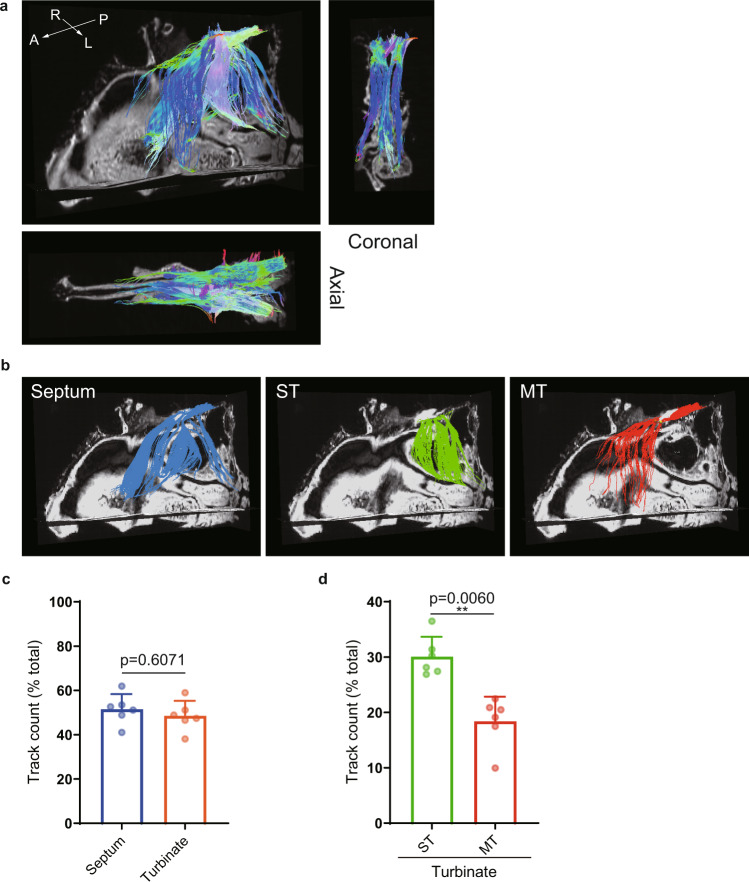
The distribution of human olfactory nerves depicted by diffusion tensor tractography (DTT). **a** DTT was performed using human nasal specimens. The reconstructed three-dimensional images show oblique-sagittal, coronal and axial views. The colored fibers were visualized with DTT, and the b0 images were used as the background images. The olfactory bulbs were selected as the region of interest (ROI) so that the depicted fibers reflected the neurons projecting from the nasal cavity to the olfactory bulbs. The fibers are color-coded according to their directionality: anterior-posterior axis, green; medial-lateral axis, red; and superior-inferior axis, blue. A anterior, L lateral, M medial, P posterior. **b** Oblique-sagittal views of the fiber tracts projecting from the main anatomical structures in the nasal cavity. The nerve tracts identified by DTT were color-coded according to the structure selected as the ROI (middle turbinate or superior turbinate). MT middle turbinate; ST superior turbinate. **c** The track counting function of TrackVis was used to measure the density of fibers distributed in the septum and turbinates. Each value is expressed as the percentage of the total track counts. Bars show the mean and standard deviation. Individual data points are shown in dots (*n* = 6; 6 nasal cavities from 3 biologically independent specimens). **d** The percentage of all track counts of the fibers distributed to each of the turbinates. MT middle turbinate, ST superior turbinate. Bars show the mean and standard deviation. Individual data points are shown in dots (*n* = 6; 6 nasal cavities from 3 biologically independent specimens). **p < 0.01.

Figure 7a depicts the nerve fibers in the nasal cavity when the olfactory bulb was set as the ROI. The fibers were distributed to the superior portion of the septum, the superior turbinate and the anterior portion of the middle turbinate. Subsequently, each of these three regions was selected as the ROI for further analysis (Fig. 7b). The fibers from the nasal septum were distributed anteriorly to the front portion of the perpendicular plate of the ethmoid bone, posteriorly to the front wall of the sphenoid sinus, and inferiorly below the lower aspect of the superior turbinate. The fibers to the middle turbinate were distributed anteriorly to a region beyond the lower end of the superior turbinate, while those to the superior turbinate were distributed throughout the structure. Track counting (with each nasal cavity considered as one sample) demonstrated that the connectivity and distribution of nerve fibers towards the septum and turbinates was comparable (Fig. 7c) and that the superior turbinate had a significantly stronger connectivity and distribution of nerve fibers than the middle turbinate (Fig. 7d).

**Fig. 8:**
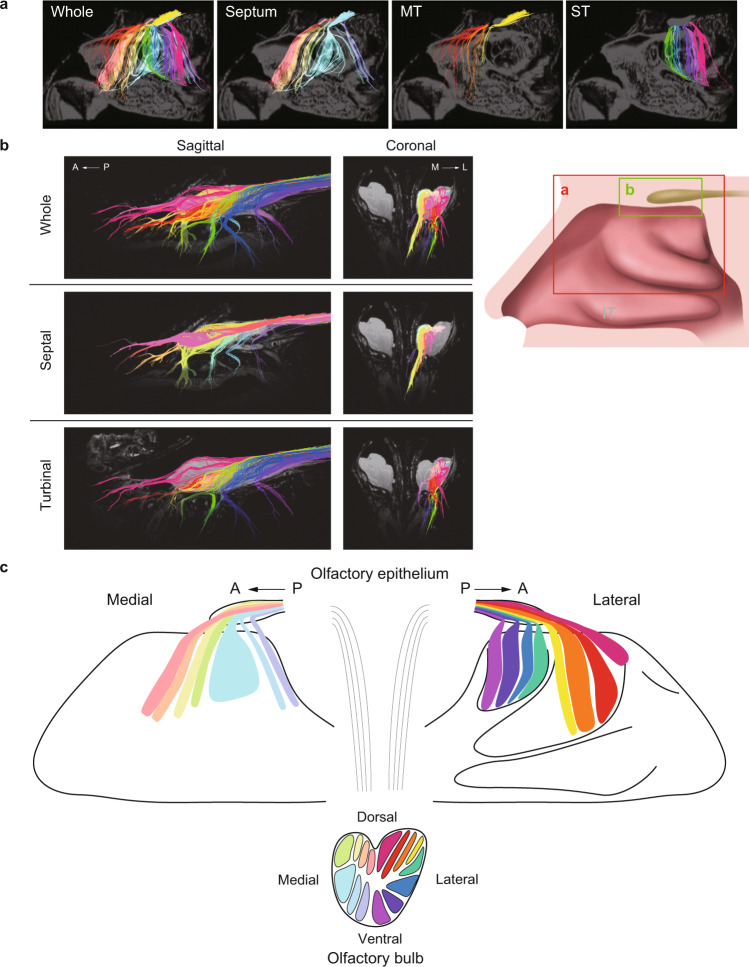
The topographical relationships of the human olfactory nerves running between the olfactory epithelium and olfactory bulb. **a** Sagittal sections depicting the nerve pathways from the olfactory epithelium to the olfactory bulb. The fibers are colored separately according to each olfactory filament. The field-of-view was limited to the red frame shown in the schema due to the restricted size of the magnetic resonance imaging system. The Whole image is a combination of the other three images. MT middle turbinate, ST superior turbinate. **b** Tractography performed with a cryoprobe. The field-of-view was limited to the green frame shown in the schema. The fibers are colored separately according to each olfactory filament. **c** Schematic diagram showing the DTT-based olfactory nerve map for the human (the distribution of the olfactory nerves running between the olfactory epithelium and olfactory bulb). Topographically, there was a correlation between the distributions in the olfactory epithelium and olfactory bulb in both the dorsal-ventral and medial-lateral axes. A anterior, P posterior.

Next, the ROI was set at each olfactory filament. The image was displayed with the colors set in a rainbow gradient from red (anterior) to purple (posterior) and with fibers on the septum depicted in light colors and those on the turbinate depicted in dark colors (Fig. 8a and Supplementary Movie 3). This color-coded DTT image revealed the distribution of nerve fibers in the nasal cavity but was not sufficiently detailed to display the projections to the olfactory bulb. Therefore, the specimen was resected 5 mm below the cribriform plate, and a cryoprobe was used to focus on the olfactory filaments passing through the cribriform plate and their projections to the olfactory bulb. Using this procedure, we were able to visualize the nerve pathways of each olfactory filament (Fig. 8b and Supplementary Movie 4). The anterior-posterior axis was preserved for both the septal and turbinal fibers (see sagittal images in Fig. 8b); nerve fibers from the septal side projected to the medial half of the olfactory bulb, while those from the turbinal side projected to the lateral half of the olfactory bulb. There was a noticeable notch in the superior surface of the olfactory bulb between the septal and turbinal fibers (see coronal images in Fig. 8b). Finally, we combined the olfactory filaments in Fig. 8a, b to generate the human olfactory nerve map showing the olfactory nerve projections from the olfactory epithelium to the olfactory bulb (Fig. 8c).

### Use of DTT to depict human olfactory nerves in a specimen from a patient who had undergone nasal surgery for chronic rhinosinusitis

Another donated human specimen was used to explore the validity of using DTT to evaluate changes in the olfactory nerves after surgery. The specimen was obtained from an 87-year-old female with olfactory nerve impairment due to nasal surgery or chronic rhinosinusitis. The specimen contained a surgical scar from an incision in the medial periorbital area that was made during external frontoethmoidectomy. Consistent with the location of the surgical scar, the honeycomb structure of the ethmoid sinus had been dissected. Notably, the tracts depicted by DTT were shorter in length for this specimen than for the other two human specimens from donors without a known history of nasal disorders or surgery (Supplementary Fig. 3).

## Discussion

In the present study, we used high-field MRI and DTT to successfully visualize the distribution of human olfactory nerves and create the human olfactory nerve map. First, we analyzed the olfactory nerve distribution depicted by DTT in mice to validate the findings against those described in existing studies. The DTT-based anatomical olfactory nerve map of the mouse, which showed the olfactory nerve projections from the nasal septum and six turbinates to the olfactory bulb, revealed that the dorsal-ventral axis and medial-lateral axis were generally preserved between the olfactory epithelium and olfactory bulb. This finding is consistent with results obtained previously using transgenic animals or tracer experiments^1,3,7,14^. Next, we performed a similar analysis using the common marmoset, which has a nasal cavity that is structurally similar to that of the human. The DTT-based olfactory nerve map of the marmoset, which was produced by depicting each olfactory filament, demonstrated that the dorsal-ventral axis and medial-lateral axis were well maintained, as in mice. Finally, we applied DTT to specimens from three donated bodies, including one post-operative sample, to depict the olfactory nerves of humans. We found that the olfactory nerves were distributed to the nasal septum, superior turbinate and lower part of the middle turbinate. Furthermore, maintenance of the dorsal-ventral and medial-lateral axes between the olfactory epithelium, olfactory bulb and olfactory tract was also observed in humans. Analysis of the post-operative sample suggested that DTT might have utility in the detection of olfactory nerve abnormalities. Based on our data, we consider DTT capable of providing an accurate assessment of olfactory nerve distribution. Further research is merited to establish whether this technique could be developed into an objective examination for olfactory dysfunction.

One major limitation of DTT is that it provides only an estimation of the neuronal pathways and does not depict each of the nerve axons. We achieved quite a high DTT resolution of 0.06 mm to 0.2 mm cubed because we used a 9.4-T scanner. However, the resolution was still not high enough to depict olfactory nerve axons, which are unmyelinated and have typical diameters less than 1 µm. Thus, DTT only detects nerve axon fascicles and not the axons themselves. Fig. 6ei, eii show that DTT identified nerve fascicles thicker than 0.2 mm but not fascicles thinner than 0.2 mm. This characteristic of the DTT technique might be expected to reduce the false-positive rate, since DTT would detect only the thicker nerve fascicles rather than structures that are not nerve fibers.

In order to have potential clinical utility as an investigation for olfactory disturbances, it is essential that DTT be shown to have specificity in the detection of olfactory nerves. Therefore, we carried out analyses to validate the DTT findings in mice and marmosets. The nerve pathways depicted by DTT in mice were verified against published data obtained using OMP-lacZ mice^1^. Furthermore, the fibers displayed by DTT in marmosets corresponded to OMP-positive regions in tissue sections, and the dorsal distribution of ACSM4-positive olfactory epithelium and its projection to the dorsal region of the olfactory bulb matched the staining pattern described in a previous report^15^. However, DTT was unable to depict thin OMP-positive nerve fibers as mentioned earlier, suggesting that the actual distribution of olfactory nerves may be wider than that visualized by DTT.

We created DTT-based olfactory nerve maps for mice, marmosets and humans, which allowed us to compare olfactory nerve distribution among the three species. Notably, the dorsal-ventral axis and medial-lateral axis were maintained in all species. Track counting revealed that mice had significantly weaker fiber connectivity and distribution towards the septum than towards the turbinates, which have more complex structures and larger surface areas than the septum. In contrast, marmosets and humans exhibited no significant differences in nerve fiber connectivity between the septum and turbinates. This latter result agrees with a previous finding that the proportion of the olfactory epithelium found on the ethmoturbinates was smaller in marmosets than in mice^11^. Additionally, both marmosets and humans had stronger nerve fiber connectivity and distribution towards the superior turbinate than towards the middle turbinate. In agreement with our findings in marmosets, experiments detecting the expression of olfactory cell adhesion molecule (a marker of ventral olfactory epithelium) in macaques confirmed that the dorsal-ventral axis is maintained for the nerve projections between the olfactory epithelium and olfactory bulb^16^. Other studies investigating olfactory nerve pathways and olfactory receptor gene expression support the existence of a topographical correlation between the olfactory epithelium and olfactory bulb^7,16–22^. Therefore, the olfactory nerve map depicted by DTT in the present study is consistent with the olfactory nerve maps reported by other studies using different approaches.

The taxonomical classifications of rodents, marmosets and humans are shown in Supplementary Fig. 4. Humans and marmosets are not only in the same order (Primates) but are also in the same suborder (Haplorhini) and infraorder (Simiiformes). Within the Simiiformes taxon, humans are in the parvorder Catarrhini, otherwise known as Old World monkeys, while marmosets are in the parvorder Platyrrhini, otherwise known as New World monkeys. These two parvorders are said to have split around 35 million years ago^23,24^ and evolved individually in separate continents. One of their most distinguishable differences is in their nasal appearance as expressed in their taxonomic name: Catarrhini meaning down-nosed (i.e., nostrils closer together and facing downward) and Platyrrhini meaning flat-nosed (i.e., nostrils wider apart and facing sideways). On the other hand, the intranasal turbinal structures of Catarrhini and Platyrrhini have many similarities such as a single maxilloturbinate and two ethmoturbinates, which is also a common feature of Haplorhines^25,26^. By contrast, many species in the suborder Strepsirrhini (e.g., lemurs and lorises) have more than four ethmoturbinates^25–27^ as well as a longer and wet nose, which often results in better olfaction. These nasal structures of Strepsirrhines are more like those seen in rodents, which typically have six ethmoturbinates^28,29^. Previous studies have also described structural differences in olfactory nerve projection between rodents and primates. For example, olfactory nerve cells expressing the same olfactory receptor gene project to only one or two glomeruli in rodents but to three or four glomeruli in marmosets^30^. The number of olfactory receptor genes also differs between rodents and primates: mice have over 1000 intact olfactory receptor genes^31,32^, whereas marmosets and humans have 393 and 396 intact olfactory receptor genes, respectively^12^. Similarities and differences between these three species are summarized^11,12,26,28,32–40^ in Table 1. Taking into consideration the similarities in olfactory receptor gene number and nasal anatomy between marmosets and humans, we suggest that marmosets^6^ may be more useful than rodents as an animal model for studies of olfaction.

**Table 1:** Summary of the similarities and differences between mice, marmosets and humans.

Feature	Mice	Marmosets	Humans
Maxilloturbinate	1	1	1
Ethmoturbinate	6	2	2
Frontal sinus	Absent	Present	Present
Maxillary sinus	Present	Present	Present
Sphenoid sinus	Absent	Present	Present
Ethmoid sinus	Multilamellar	Multicellular	Multicellular
Vomeronasal organ	Present	Present	Present in fetus, debatable in adults
Intact olfactory receptor genes	1036–1223	383–393	323–396
Mean track count ratio (septum: turbinates)	3: 7	5: 5	5: 5

To the best of our knowledge, the present study is the first to generate a DTT-based olfactory nerve map that clarifies the distribution of olfactory nerves in the nasal cavity of humans. Although it was our original intention to perform a histological analysis to validate the DTT-based findings, this was not achievable because the human samples were not well-preserved and the epithelium became detached during sectioning. Nevertheless, it is possible to compare the distribution of olfactory nerves obtained in this study with that described in previous reports. Most published investigations describing the distribution of nerves in human olfactory epithelium are based on biopsies, precluding a comprehensive analysis. In addition, there are ethical issues regarding the use of human biopsy tissue for research purposes, which limits the use of this approach. Histological analyses of the entire olfactory epithelium require the use of a cadaver, but only a few such investigations have been performed. The majority of these studies concluded that olfactory nerves are present in the middle turbinate. One study reported that olfactory nerves in the middle turbinate were present up to an average of 15.3 mm below the cribriform plate^41^, a second study identified olfactory nerve endings up to 7–20 mm below the cribriform plate^42^, and a third investigation found that the OMP-positive region extended up to the lower portion of the middle turbinate^43^. Despite some variation between studies regarding the distribution of the olfactory epithelium, most histological analyses have demonstrated that olfactory nerves are present in the mucosa of the middle turbinate near its attachment to the lateral nasal wall^44–48^. One interesting study using electroolfactography found that 7 out of 12 human subjects exhibited a response to vanillin, with some of the subjects also showing a response at the anterior wall of the middle turbinate^49^. Our DTT-based data are consistent with this result since olfactory nerves were depicted in the anterior part of the middle turbinate. Additionally, we calculated that the lengths of the olfactory nerves projecting from the middle turbinate ranged from 8–22 mm, which is consistent with previous reports measuring the extent to which the olfactory mucosa or nerve endings extended beyond the cribriform plate^41,42^. A recent study of human cadavers^50^ reported that the olfactory epithelial surface area in the nasal septum, middle turbinate and upper turbinate was 173 mm^2^, 77 mm^2^ and 96 mm^2^, respectively, and we observed a similar trend when the track count was compared between these structures (Fig. 7). Overall, our results suggest that around 20% of the olfactory nerves are distributed to the middle turbinate (a higher proportion than previously thought), implying that this structure could play an important role in olfaction.

Our findings indicate that DTT potentially could be used in clinical practice to diagnose and evaluate olfactory dysfunction and assist in the planning of surgical procedures. Olfactory deficits are mainly evaluated by psychophysical olfactory assessments^51,52^, but these investigations provide subjective evaluations that are not suitable for inter-individual comparisons and cannot exclude the possibility of malingering. Additionally, psychophysical assessments give little insight into the type of olfactory dysfunction (conductive, sensorineural or central) or the anatomical location of the lesion. DTT is a neuroimaging technique that enables the visualization of pathways and estimation of nerve fiber thickness. DTT has already been applied clinically in the field of neurosurgery, where it is useful for the preoperative evaluation of brain tumors^53,54^. If it becomes possible to apply this technique to the nasal cavity, DTT potentially could be used to identify the sites of olfactory nerve damage and thereby facilitate the diagnosis of olfactory disorders. Notably, in the present study, the nerve fibers depicted by DTT were shorter for the specimen from the patient who had nasal surgery for chronic rhinosinusitis than for the two human specimens from donors without a known history of nasal disorders or surgery. This shortening of the nerve fibers may have been caused by the surgical procedure, chronic rhinosinusitis or both. Although a formal statistical analysis could not be performed due to the small number of available specimens, the results indicate that DTT has potential utility in the detection of olfactory neuropathy. Furthermore, DTT-based track counting could be utilized as an objective quantitative test in the evaluation of olfactory disorders. Additionally, the first olfactory fiber is sometimes used as an anatomical landmark in endoscopic sinus surgery for chronic sinusitis^55^. Our intranasal olfactory nerve map might provide information to facilitate surgical procedures such as septoplasty and endoscopic sinus surgery. There are still many hurdles to overcome before DTT can be applied in clinical practice to patients with olfactory dysfunction, such as the need to increase the magnetic field and develop a dedicated coil. Furthermore, the scan time will need to be shortened from days to minutes in order for DTT to be suitable for use in patients, which may require the development of new software for image enhancement and fiber tracking. Our research provides a basis for the establishment of an objective method of evaluating the olfactory nerves using DTT.

## Methods

### Ethics

All experimental procedures were performed at The Jikei University in Japan. Animal protocols were approved by the Institutional Animal Care and Use Committee of The Jikei University (approval number: 2020-002 and 2020-006) and performed in accordance with the guidelines of the National Institutes of Health and the Ministry of Education, Culture, Sports, Science and Technology of Japan. All experimental protocols using human samples in this study were approved by the Ethics Committee of The Jikei University School of Medicine (approval number: 26-001 7506). This study conformed to the guidelines of the Declaration of Helsinki and informed consent was obtained for human specimens.

### Animals

The present study used the heads of three mice (strain: C57BL/6NCrSlc; age: 4 weeks old; Japan SLC, Shizuoka, Japan) and five common marmosets (strain: EDM: C. Marmoset (Jic); age: 2–3 years old; CLEA Japan, Tokyo, Japan) that had been fixed with 4% paraformaldehyde in phosphate-buffered saline for 2 weeks. After MRI scanning of each marmoset head, the nasal cavity was carefully dissected out and prepared as a paraffin-embedded block for subsequent histological analysis.

### Human samples

Donated human cadaveric bodies (a 79-year-old male, an 81-year-old female and an 87-year-old female) were fixed with formol-carbol. The causes of death were lung cancer for the 79-year-old male, gastric cancer for the 81-year-old female and pancreatic cancer for the 87-year-old female. The cadaveric bodies were carefully dissected by three board-certified otorhinolaryngologists (S.K., M.T. and E.M.) to obtain the nasal cavities with the olfactory bulbs attached.

### MRI protocols

The specimens were fixed and stored for 2 weeks in phosphate-buffered saline containing 1 mM Magnevist (Bayer AG, Leverkusen, Germany)^56^ to improve the signal-to-noise ratio. Specimens were scanned using a 9.4-T MRI scanner (Bruker BioSpin MRI GmbH, Ettlingen, Germany) with a bore diameter of 200 mm and a maximum gradient strength of 300 mT/m on each axis. A cryogenic quadrature radiofrequency surface probe (CryoProbe, Bruker BioSpin AG, Fällanden, Switzerland) was used for human and mouse specimens, and an 86-mm volume coil (Bruker BioSpin MRI GmbH, Ettlingen, Germany) was utilized for human and marmoset specimens.

Diffusion-weighted imaging data were acquired using a 3D spin-echo sequence based on a Stejskal-Tanner diffusion preparation. The scanning parameters were as follows: repetition time, 500 ms; echo time, 27.8 ms; flip angle, 90°; field-of-view, 56.7 mm × 80.5 mm × 26.3 mm (humans, volume coil), 21.6 mm × 8.8 mm × 13.4 mm (humans, cryoprobe), 30 mm × 24 mm × 14 mm (marmosets) or 22 mm × 8 mm × 12 mm (mice); acquisition data matrix, 165 × 230 × 75 (humans, volume coil), 360 × 146 × 222 (humans, cryoprobe), 150 × 120 × 70 (marmosets) or 250 × 92 × 136 (mice); reconstructed image resolution, 0.35 mm × 0.35 mm × 0.35 mm (humans, volume coil), 0.06 mm × 0.06 mm × 0.06 mm (humans, cryoprobe), 0.2 mm × 0.2 mm × 0.2 mm (marmosets) or 0.088 mm × 0.088 mm × 0.088 mm (mice); b-value, 3,000 s/mm^2^; motion-probing gradient orientations, 30 axes; motion-probing gradient duration/separation, 6/12 ms; number of repetitions used for signal averaging, 1 (humans and mice) or 3 (marmosets); and scan time, 77 h (humans, volume coil), 71 h (humans, cryoprobe), 59 h (marmosets) or 58 h (mice). Imaging data were denoised using MRtrix3 (www.mrtrix.org)^57,58^. Correction for distortion was not performed since we used a conventional acquisition method that acquires one row at a time in k-space with little distortion. B0 images were used for the anatomical images. DTT was performed by analyzing the acquired diffusion-weighted MRI data using Diffusion Toolkit software (Massachusetts General Hospital, Boston, MA, USA)^59^. Visualization of the fiber tracts from the obtained DTT data was carried out using TrackVis software (Massachusetts General Hospital, Boston, MA, USA)^59^. Tractography was conducted based on fiber assignment with a continuous tracking algorithm^60^ by tracking the principal eigenvector (e1) in each voxel and reconstructing it in 3D. Thus, all fiber tracts contained in each specimen were displayed. We then applied multiple ROIs to selectively display fiber tracts for individual anatomical structures^61^. Tractography was performed bilaterally for each specimen (two specimens for humans, five for marmosets and three for mice).

### Histology and immunohistochemistry

After the completion of MRI, the samples were dissected, embedded in paraffin blocks and sectioned at a thickness of 4–6 μm. Histological analyses were performed after staining of the sections with hematoxylin and eosin using standard procedures. For immunohistochemistry, the sections were treated with proteinase K for antigen retrieval and then blocked with skim milk for 1 h at 25 °C. Then, the sections were incubated overnight at 4 °C with the following primary antibodies: rabbit polyclonal anti-ACSM4 antibody (1:100; HPA049895, Sigma–Aldrich, St. Louis, MO, USA) or goat polyclonal anti-OMP antibody (1:500; 019-22291, Wako Pure Chemical Industries, Osaka, Japan). The sections were rinsed with Tris-buffered saline and incubated with biotinylated secondary antibodies conjugated to streptavidin-horseradish peroxidase. The chromogenic reaction was produced by the addition of diaminobenzidine tetrahydrochloride, after which the slides were immersed in distilled water to quench the reaction. The slides were counterstained with hematoxylin.

### Statistics and reproducibility

Data are expressed as the mean with standard deviation. Statistical analyses were performed using Prism 7 (GraphPad Software, La Jolla, CA, USA). Comparisons between two groups were made using Student’s *t*-test, and comparisons between three or more groups were made using one-way analysis of variance and the Tukey-Kramer post-hoc test. A *p*-value < 0.05 was considered statistically significant. Biological replicates were performed using heads of three mice, five common marmosets and nasal cavities of three donated human cadaveric bodies. MRI scans were repeated three times for marmosets and one time for humans and mice for signal averaging.

### Reporting summary

Further information on research design is available in the Nature Research Reporting Summary linked to this article.

## Supplementary information

Supplementary Information

Description of Additional Supplementary Files

Supplementary Data 1

Supplementary Data 2

Supplementary Data 3

Supplementary Movie 1

Supplementary Movie 2

Supplementary Movie 3

Supplementary Movie 4

Reporting Summary

## Data Availability

Source data underlying Figs. 1c, d, 4c, d and 7c, d are provided in Supplementary Data 1, 2 and 3, respectively. Other data that support the findings of this study are available from the corresponding author upon reasonable request.
