# Author Correction: MRI tractography reveals the human olfactory nerve map connecting the olfactory epithelium and olfactory bulb

**DOI:** 10.1038/s42003-022-04220-z

**Published:** 2022-11-10

**Authors:** Sho Kurihara, Masayoshi Tei, Junichi Hata, Eri Mori, Masato Fujioka, Yoshinori Matsuwaki, Nobuyoshi Otori, Hiromi Kojima, Hirotaka James Okano

**Affiliations:** 1 Department of Otorhinolaryngology, The Jikei University School of Medicine, 3-25-8 Nishishimbashi Minato-ku, Tokyo, 105-8471 Japan; 2 Division of Regenerative Medicine, The Jikei University School of Medicine, 3-25-8 Nishishimbashi Minato-ku, Tokyo, 105-8471 Japan; 3 Graduate School of Human Health Sciences, Tokyo Metropolitan University, 7-2-10 Higashi-Ogu Arakawa-ku, Tokyo, 116-8551 Japan; 4 Department of Molecular Genetics, Kitasato University School of Medicine, 1-15-1 Kitasato Minami-ku Sagamihara-shi, Kanagawa, 252-0373 Japan; 5 Department of Otorhinolaryngology, Head and Neck Surgery, Keio University School of Medicine, 35 Shinanomachi Shinjuku-ku, Tokyo, 160-8582 Japan

**Keywords:** Olfactory bulb, Neural circuits, Olfactory bulb, Diffusion tensor imaging

Correction to: *Communications Biology* 10.1038/s42003-022-03794-y, published online 6 September 2022.

The original version of this Article contained an error in the Methods subsection, “Ethics”, stating:

“All experimental protocols using human samples in this study were approved by the Ethics Committee of The Jikei University School of Medicine (approval number: 25–180 7315)”.

The “Ethics” subsection should instead state:

“All experimental protocols using human samples in this study were approved by the Ethics Committee of The Jikei University School of Medicine (approval number: 26-001 7506)”.

This error has now been corrected in the PDF and HTML versions of the Article.

