# Supplementary material for: MRI tractography reveals the human olfactory nerve map connecting the olfactory epithelium and olfactory bulb
Source: Commun Biol. 2022 Sep 6;5:843. doi: 10.1038/s42003-022-03794-y (PMC9448749; doi:10.1038/s42003-022-03794-y)
Supplement: Supplementary file 1 — Supplementary Information [file 42003_2022_3794_MOESM1_ESM.pdf]

Supplementary Fig. 1: Intranasal tissue from P2-IRES-tau-lacZ mice stained with X-Gal.

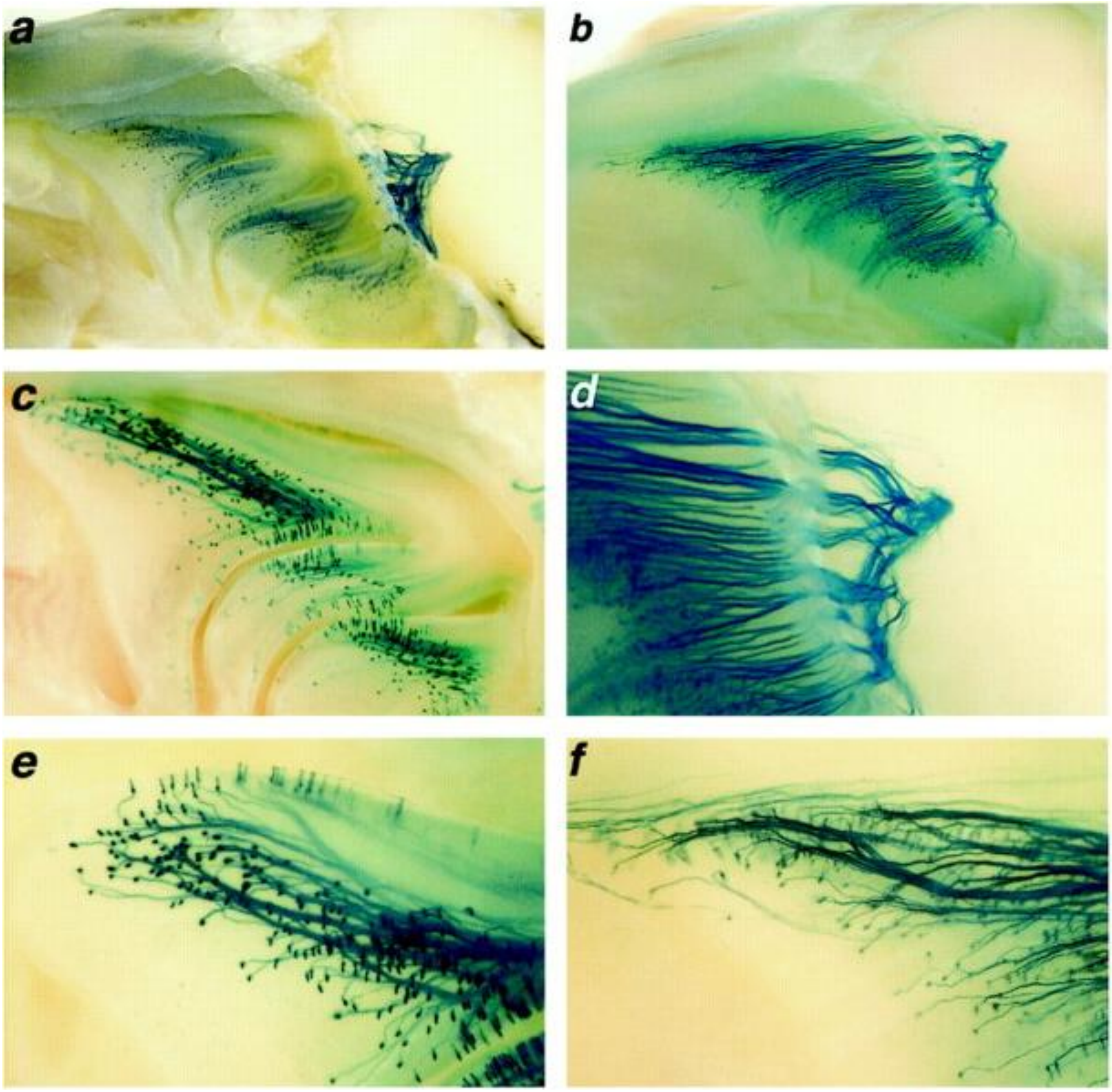

Figure reprinted from Mombaerts et al., 1996[1]. Olfactory nerve cells (dendrites, cell bodies and axons) are stained blue with X-Gal.

Supplementary Fig. 2: Original images corresponding to those shown in Figure 6.

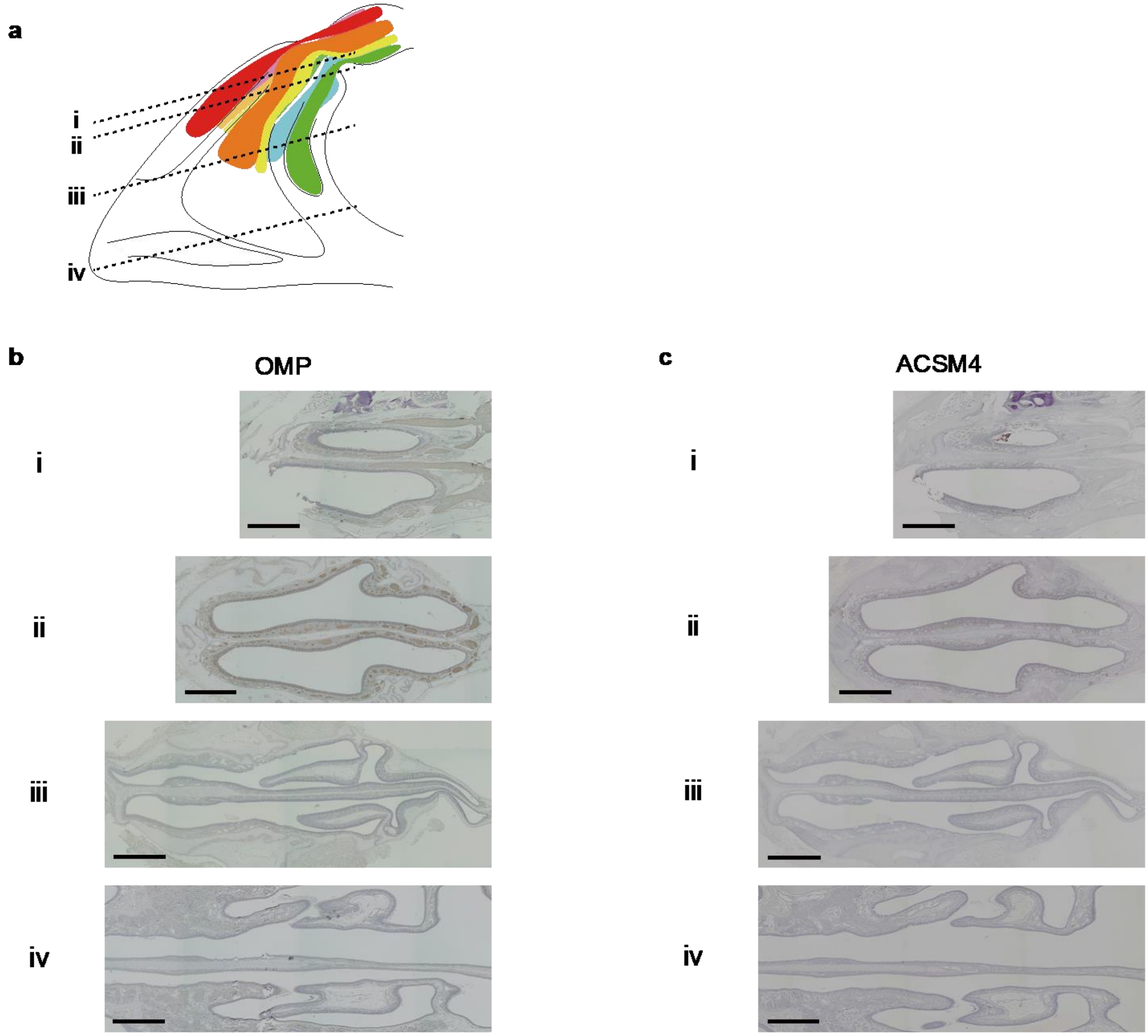

a Tissue sections were obtained at four levels (i, ii, iii and iv). **b** Histological sections of the marmoset nasal cavity immunostained for olfactory marker protein (OMP). In addition to the positively-staining regions in the mucosal layer, nerve bundles can be seen in the positively-stained regions in the submucosal layer. Scale bars: 1 mm. **c** Histological sections of the marmoset nasal cavity immunostained for acyl-coenzyme A synthetase medium chain family member-4 (ACSM4). The positively-staining regions were limited to the mucosal layer and were less extensive than the OMP-positive regions. Scale bars: 1 mm.

Supplementary Fig. 3: Diffusion tensor tractography (DTT) of a human specimen from a patient who had undergone surgery for chronic rhinosinusitis.

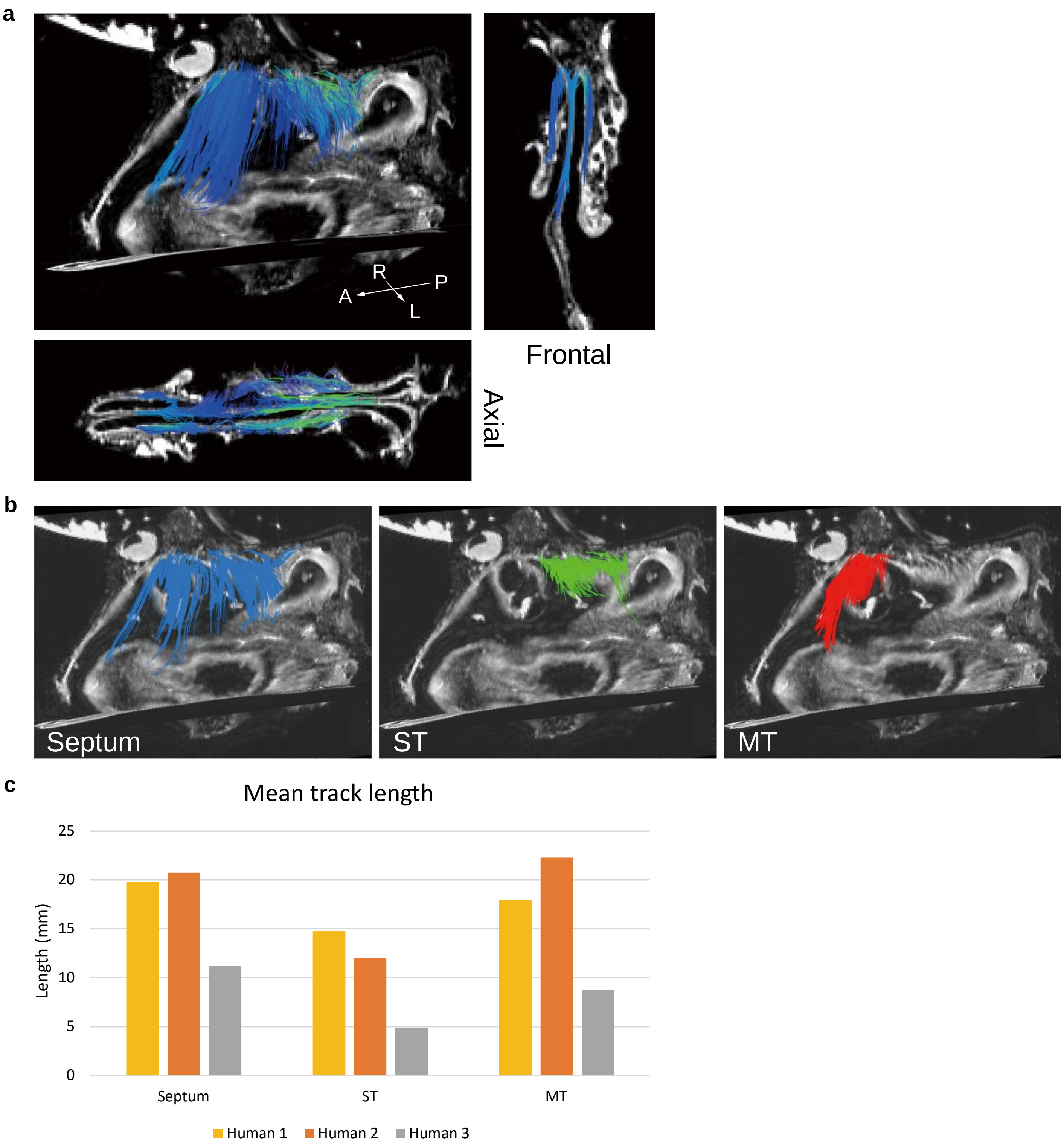

a DTT was performed using a human specimen obtained from a patient who had undergone surgery for chronic rhinosinusitis. The reconstructed three-dimensional images show oblique-sagittal, coronal and axial views. The colored fibers were visualized with DTT, and the b0 images were used as the background images. The olfactory bulbs were selected as the region of interest (ROI) so that the depicted fibers reflected the neurons projecting from the nasal cavity to the olfactory bulbs. The fibers are color-coded according to their directionality: anterior-posterior axis, green; medial-lateral axis, red; and superior-inferior axis, blue. A, anterior; L, lateral; M, medial; P, posterior. **b** Oblique-sagittal views of the fiber tracts projecting from the main anatomical structures in the nasal cavity. The nerve tracts identified by DTT were color-coded according to the structure selected as the ROI (middle turbinate or superior turbinate). MT middle turbinate; ST, superior turbinate. **c** The mean track length for the three human specimens. The post-operative specimen (Human 3) had a shorter track length compared to the other two human specimens (Human 1 and Human 2).

Supplementary Fig. 4: Taxonomy chart for the three species.

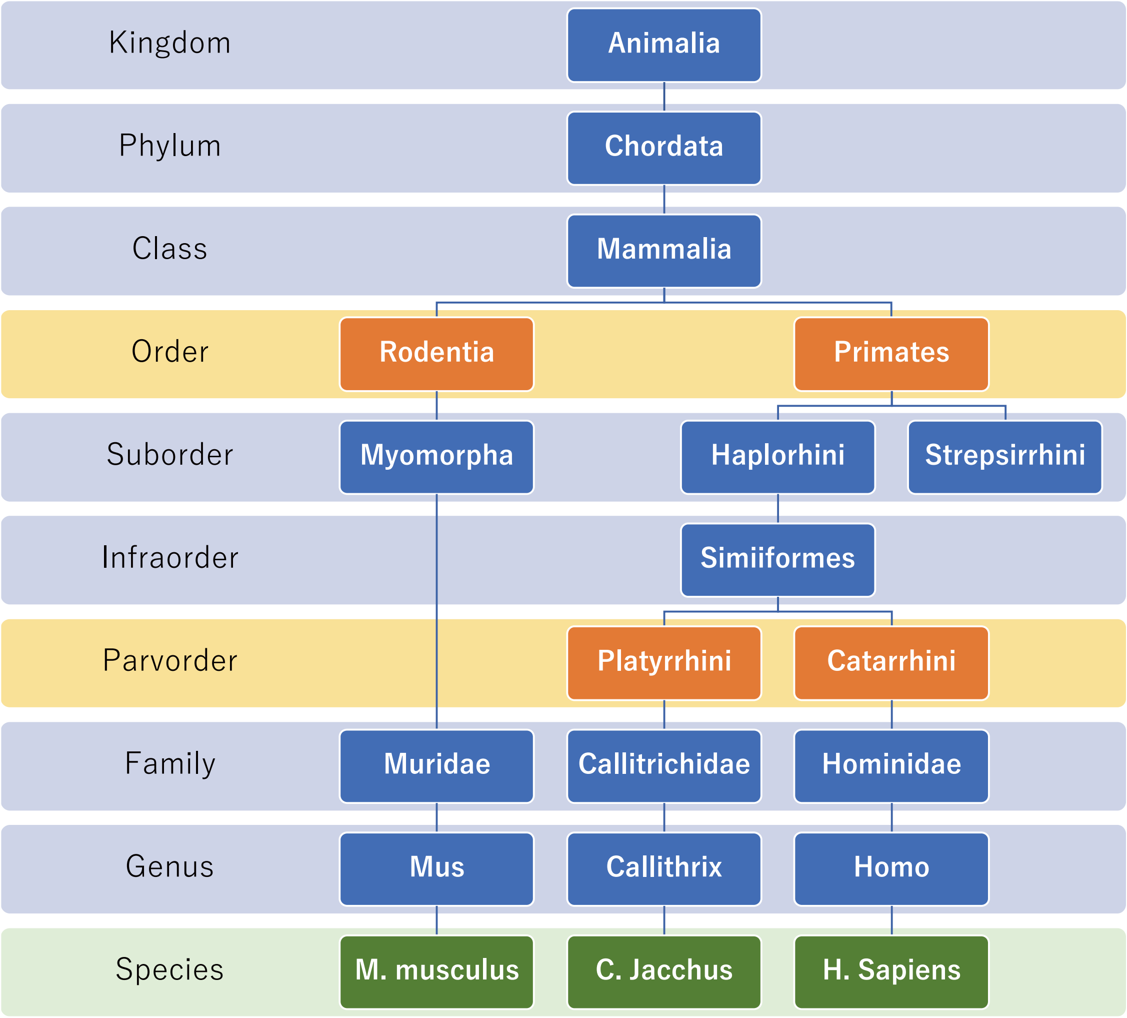

M. musculus: mouse, C. Jacchs: marmoset, H. sapiens: human.

Supplementary References

1 Mombaerts, P. *et al.* Visualizing an olfactory sensory map. *Cell* **87**, 675-686, https://doi.org/10.1016/s0092-8674(00)81387-2 (1996).
