# Supplementary material for: MRI tractography reveals the human olfactory nerve map connecting the olfactory epithelium and olfactory bulb
Source: Commun Biol. 2022 Sep 6;5:843. doi: 10.1038/s42003-022-03794-y (PMC9448749; doi:10.1038/s42003-022-03794-y)
Supplement: Supplementary file 2 — Description of Additional Supplementary Files [file 42003_2022_3794_MOESM2_ESM.pdf]

Description of Additional Supplementary Files

File name: Supplementary Data 1
Description: Source data underlying Fig 1c-d mouse track count

File name: Supplementary Data 2
Description: Source data underlying Fig 4c-d marmoset track count

File name: Supplementary Data 3
Description: Source data underlying Fig 7c-d human track count

File name: Supplementary Movie 1
Description: Rotating three-dimensional reconstruction of the mouse nasal cavity obtained by diffusion tensor tractography.

File name: Supplementary Movie 2
Description: Rotating three-dimensional reconstruction of the marmoset nasal cavity obtained by diffusion tensor tractography.

File name: Supplementary Movie 3
Description: Rotating three-dimensional reconstruction of the human nasal cavity obtained by diffusion tensor tractography.

File name: Supplementary Movie 4
Description: Rotating three-dimensional reconstruction of the human olfactory bulb obtained by diffusion tensor tractography.
