# Supplementary material for: MRI tractography reveals the human olfactory nerve map connecting the olfactory epithelium and olfactory bulb
Source: Commun Biol. 2022 Sep 6;5:843. doi: 10.1038/s42003-022-03794-y (PMC9448749; doi:10.1038/s42003-022-03794-y)
Supplement: Supplementary file 10 — Reporting Summary [file 42003_2022_3794_MOESM10_ESM.pdf]

nature portfolio

Corresponding author(s): Sho Kurihara

Last updated by author(s): Jul 1, 2022

Reporting Summary

Nature Portfolio wishes to improve the reproducibility of the work that we publish. This form provides structure for consistency and transparency in reporting. For further information on Nature Portfolio policies, see our Editorial Policies and the Editorial Policy Checklist.

Statistics

For all statistical analyses, confirm that the following items are present in the figure legend, table legend, main text, or Methods section.

n/a	Confirmed	
☐	☒	The exact sample size (*n*) for each experimental group/condition, given as a discrete number and unit of measurement
☐	☒	A statement on whether measurements were taken from distinct samples or whether the same sample was measured repeatedly
☐	☒	The statistical test(s) used AND whether they are one- or two-sided<br>*Only common tests should be described solely by name; describe more complex techniques in the Methods section.*
☒	☐	A description of all covariates tested
☒	☐	A description of any assumptions or corrections, such as tests of normality and adjustment for multiple comparisons
☐	☒	A full description of the statistical parameters including central tendency (e.g. means) or other basic estimates (e.g. regression coefficient) AND variation (e.g. standard deviation) or associated estimates of uncertainty (e.g. confidence intervals)
☐	☒	For null hypothesis testing, the test statistic (e.g. *F*, *t*, *r*) with confidence intervals, effect sizes, degrees of freedom and *P* value noted<br>*Give P values as exact values whenever suitable.*
☒	☐	For Bayesian analysis, information on the choice of priors and Markov chain Monte Carlo settings
☒	☐	For hierarchical and complex designs, identification of the appropriate level for tests and full reporting of outcomes
☒	☐	Estimates of effect sizes (e.g. Cohen's *d*, Pearson's *r*), indicating how they were calculated

Our web collection on statistics for biologists contains articles on many of the points above.

Software and code

Policy information about availability of computer code

Data collection	Diffusion Toolkit software (Massachusetts General Hospital, Boston, MA, USA), TrackVis software (Massachusetts General Hospital, Boston, MA, USA)
Data analysis	TrackVis software (Massachusetts General Hospital, Boston, MA, USA), Prism 7 (GraphPad Software, La Jolla, CA, USA)

For manuscripts utilizing custom algorithms or software that are central to the research but not yet described in published literature, software must be made available to editors and reviewers. We strongly encourage code deposition in a community repository (e.g. GitHub). See the Nature Portfolio guidelines for submitting code & software for further information.

Data

Policy information about availability of data

All manuscripts must include a data availability statement. This statement should provide the following information, where applicable:

- Accession codes, unique identifiers, or web links for publicly available datasets
- A description of any restrictions on data availability
- For clinical datasets or third party data, please ensure that the statement adheres to our policy

Source data underlying Fig 1c-d, Fig 4c-d and Fig 7c-d are provided in Supplementary Data 1, 2 and 3, respectively. Other data that support the findings of this study are available from the corresponding author upon reasonable request.

Field-specific reporting

Please select the one below that is the best fit for your research. If you are not sure, read the appropriate sections before making your selection.

☒ Life sciences ☐ Behavioural & social sciences ☐ Ecological, evolutionary & environmental sciences

For a reference copy of the document with all sections, see nature.com/documents/nr-reporting-summary-flat.pdf

Life sciences study design

All studies must disclose on these points even when the disclosure is negative.

Sample size	Cadaveric heads of three mice, five common marmosets and three humans were used to confirm reproducibility of our analyses and to perform statistical analyses including the Student's t-test and the Tukey-Kramer post-hoc test.
Data exclusions	No data were excluded.
Replication	Cadaveric heads of three mice, five common marmosets and three humans were used to confirm reproducibility of our analyses.
Randomization	Not applicable for our analyses.
Blinding	Not applicable for our analyses.

Reporting for specific materials, systems and methods

We require information from authors about some types of materials, experimental systems and methods used in many studies. Here, indicate whether each material, system or method listed is relevant to your study. If you are not sure if a list item applies to your research, read the appropriate section before selecting a response.

Materials & experimental systems

n/a	Involved in the study	
☐	☒	Antibodies
☒	☐	Eukaryotic cell lines
☒	☐	Palaeontology and archaeology
☐	☒	Animals and other organisms
☒	☐	Human research participants
☒	☐	Clinical data
☒	☐	Dual use research of concern

Methods

n/a	Involved in the study	
☒	☐	ChIP-seq
☒	☐	Flow cytometry
☐	☒	MRI-based neuroimaging

Antibodies

Antibodies used	Anti-ACSM4 antibody (HPA049895, Sigma-Aldrich, St. Louis, MO, USA), Anti-OMP antibody (019-22291, Wako Pure Chemical Industries, Osaka, Japan)
Validation	Anti-ACSM4 Antibody (NBP2-14696), Anti-Olfactory Marker Protein (ab87338)

Animals and other organisms

Policy information about studies involving animals; ARRIVE guidelines recommended for reporting animal research

Laboratory animals	Mice (strain: C57BL/6NCrSlc; age: 4 weeks old; Japan SLC, Shizuoka, Japan), common marmosets (strain: EDM: C. Marmoset (Jic); age: 2–3 years old; CLEA Japan, Tokyo, Japan)
Wild animals	This study did not involve wild animals.
Field-collected samples	This study did not involve samples collected with field.
Ethics oversight	All experimental procedures were performed at The Jikei University in Japan. Animal protocols were approved by the Institutional Animal Care and Use Committee of The Jikei University (approval number: 2020-002 and 2020-006) and performed in accordance with the guidelines of the National Institutes of Health and the Ministry of Education, Culture, Sports, Science and Technology of Japan. All experimental protocols using human samples in this study were approved by the Ethics Committee of The Jikei University School of Medicine (approval number: 25–180 7315). This study conformed to the guidelines of the Declaration of Helsinki.

Note that full information on the approval of the study protocol must also be provided in the manuscript.

Magnetic resonance imaging

Experimental design

Design type	Scanning of specimens
Design specifications	Diffusion tensor imaging
Behavioral performance measures	Not applicable for our analyses.

Acquisition

Imaging type(s)	Diffusion tensor imaging.
Field strength	9.4 tesla
Sequence & imaging parameters	repetition time, 500 ms; echo time, 27.8 ms; flip angle, 90º; field-of-view, 56.7 mm × 80.5 mm × 26.3 mm (humans, volume coil), 21.6 mm × 8.8 mm × 13.4 mm (humans, cryoprobe), 30 mm × 24 mm × 14 mm (marmosets) or 22 mm × 8 mm × 12 mm (mice); acquisition data matrix, 165 × 230 × 75 (humans, volume coil), 360 × 146 × 222 (humans, cryoprobe), 150 × 120 × 70 (marmosets) or 250 × 92 × 136 (mice); reconstructed image resolution, 0.35 mm × 0.35 mm × 0.35 mm (humans, volume coil), 0.06 mm × 0.06 mm × 0.06 mm (humans, cryoprobe), 0.2 mm × 0.2 mm × 0.2 mm (marmosets) or 0.088 mm × 0.088 mm × 0.088 mm (mice); b-value, 3,000 s/mm2; motion-probing gradient orientations, 30 axes; motion-probing gradient duration/separation, 6/12 ms; number of repetitions used for signal averaging, 1 (humans and mice) or 3 (marmosets); and scan time, 77 hours (humans, volume coil), 71 hours (humans, cryoprobe), 59 hours (marmosets) or 58 hours (mice).
Area of acquisition	Nasal cavity

Diffusion MRI ☒ Used ☐ Not used

Parameters: b-value, 3,000 s/mm2

Preprocessing

Preprocessing software	Diffusion ToolKit
Normalization	Not applicable for our analyses.
Normalization template	Not applicable for our analyses.
Noise and artifact removal	Not applicable for our analyses.
Volume censoring	Not applicable for our analyses.

Statistical modeling & inference

Model type and settings	Not applicable for our analyses.
Effect(s) tested	Not applicable for our analyses.

Specify type of analysis: ☐ Whole brain ☒ ROI-based ☐ Both

Anatomical location(s): Nasal cavity.

Statistic type for inference (See Eklund et al. 2016)	Not applicable for our analyses.
Correction	Not applicable for our analyses.

Models & analysis

n/a	Involved in the study
☒	☐ Functional and/or effective connectivity
☒	☐ Graph analysis
☒	☐ Multivariate modeling or predictive analysis
